# Combination of Cable Cerclage and Hook Plate for the Fixation of Comminuted Fractures of Inferior Patellar Pole: A Review of 16 Consecutive Patients Followed Up for a Minimum of 1 Year

**DOI:** 10.1111/os.13481

**Published:** 2022-10-07

**Authors:** Hangyu Gu, Shiwen Zhu, Ting Li, Xinbao Wu

**Affiliations:** ^1^ Department of Traumatology and Orthopaedic Surgery Beijing Jishuitan Hospital, Fourth Clinical College of Peking University Beijing China

**Keywords:** cerclage, hook plate, inferior patellar pole, osteosynthesis, outcome, patellar fractures

## Abstract

**Objectives:**

To present a new method consisting of cable cerclage and hook plate for fixating the comminuted inferior patellar pole fracture and evaluate the outcomes.

**Methods:**

A total of 16 consecutive patients who were treated with the construct of a cable cerclage in combination with a hook plate between January 2018 and September 2020 were included in the study. Mechanism of injury, duration, and technical details of the operation were reviewed. Plain radiographs and computerized tomography (CT) scans were routinely taken to evaluate the fracture pattern. The primary outcome measures included bony healing time, pain intensity‐numerical rating scale (PI‐NRS), range of motion (ROM), and the Bostman score at the final follow‐up.

**Results:**

Eight males and eight females with an average age of 55.6 ± 12.0 years (range, 41 to 73 years) were included. Bony union was achieved in all the patients, with an average healing time of 10.8 ± 2.4 weeks (range, 8–16 weeks). With the average follow‐up of 20.1 ± 5.3 months, 12 patients (75%) had no pain (PI‐NRS score of 0), and the remaining four patients (25%) reported mild pain (three with a PI‐NRS score of 1 and one with a score of 2). The final Bostman score was 27.8 ± 3.0 (range, 20–30) on average, and all the patients showed excellent or good results. The average range of motion was 127.5° ± 13.9° (range, 90°–140°). No implant failure or hardware irritation was found during the follow‐up.

**Conclusion:**

The fixation of cable cerclage combined with hook plate resulted as a reliable method for managing the inferior patellar pole fractures, allowing immediate rehabilitation and weight‐bearing.

## Introduction

Inferior patellar pole fracture is considered to have a special pattern, accounting for 9.3% to 22.4% of all the surgically managed patellar fractures.^1^ The most common mechanisms causing such fractures are a direct hit to the flexed knee and the simultaneously forceful contraction of the quadriceps muscle that occurs instinctively.^2^ Because of the mechanism, avulsion force and direct blow cause the inferior patellar pole fragment to comminute. Larazo *et al*.^3^ reported a substantial prevalence of inferior patellar pole comminution (88%) visible on computerized tomography (CT) scan images that could not be well visualized with plain radiographs alone. Moreover, Carpenter *et al*.^4^ reported that a vertical length of the inferior patellar pole was usually less than 14 mm.

In the past decades, partial patellectomy has been used in clinical practice as an option to manage the inferior patellar pole fracture. However, partial patellectomy always provides poor results because of increased risks of osteoarthritis and patellar baja.^5^, ^6^ Therefore, it should be avoided and bone preserving procedures are recommended in current clinical practice.^7^ Although there are many surgical techniques have been reported by surgeons around the world, none are currently considered as the golden standard for the fixation of inferior patellar pole fracture. Because of the small size of the fragment and comminuted fracture pattern, it is hard to find an ideal treatment that would consistently yield positive results. For example, as the most commonly used method for the fixation of transverse patellar fractures, the tension band technique might not achieve adequate stability in managing inferior patellar pole fracture, because it requires intact and sufficient cortical bone at the compressive side. Therefore, some surgeons have modified the tension band by adding the augment of miniplate or cable cerclage.^8^, ^9^, ^10^ However, surgeons have to consider problems such as wire breakage, Kirschner wire migration, and hardware irritation.^11^, ^12^ The other commonly used and classical method is basket plate,^13^ which could preserve the bone of inferior patellar pole and provide promising outcome, but such special designed implants might not be available in some institutes.

Plate fixation could be considered as an effective technique that overcomes the shortcomings of tension band wiring.^14^, ^15^, ^16^ Biomechanical studies have demonstrated that this method is more effective than tension band constructs.^14^, ^15^, ^16^ Moreover, there are some published studies with successful experience of hook plates being used to manage avulsion fractures,^17^, ^18^, ^19^ but most of the cases are suffered from phalangeal fractures. Therefore, it is meaningful to design a convenient and effective construct by combination of hook plate fixation with cable cerclage augmentation for the management of inferior patellar pole fractures.

This study aimed to (i) introduce the surgical technique of fixation of inferior patellar pole with a combination construct of cable cerclage and hook plate and (ii) evaluate the effectiveness and safety of this technique, in other words, whether it could provide enough stability to allow early rehabilitation and get satisfactory outcomes.

## Methods

### Inclusion and Exclusion Criteria

The inclusion criteria were defined as: (i) isolated inferior patellar pole fracture (AO/OTA 34‐A1) by X‐ray and CT scan; (iii) the fracture was fixated by the construct of a cable cerclage in combination with a hook plate; (iii) at least 1 year follow‐up.

The exclusion criteria were defined as: (i) patients with systemic comorbidities which required the use of corticosteroid or compromised the knee function, such as autoimmune disease, rheumatoid arthritis, renal failure with dialysis, hemiplegia, or severe Parkinson disease, etc.; (ii) pre‐existing surgical procedure in the affected knee joint, such as total knee arthroplasty, revision surgery, etc.

### General Information of Patients

This retrospective cohort study included 16 consecutive patients with displaced inferior patellar pole fractures (AO/OTA 34‐A1) surgically fixated by a combined method with cable cerclage and hook plate between January 2018 and September 2020.

The technique was performed by qualified orthopaedic surgeons in the Department of Traumatology. There were eight males and eight female patients with an average age of 55.6 ± 12.0 years (range, 41 to 73 years); there were seven cases with a fracture on the left side and nine patients with a right‐side fracture. All the fractures resulted from falling to the ground. Also, all the surgeries were performed within 14 days from the time the fracture happened. Fifteen cases were closed type, while one case was Gustillo I open fracture; all of them were comminuted fractures visible on CT scans (Table 1).

**TABLE 1:** Patient demographics and clinical outcomes

Patient	Age (years)	Sex	Comorbidity	Mechanism of injury	Side	Type	Surgery time (min)	Follow ‐up time (months)	Bony union time (weeks)	PI‐NRS^a^	ROM^b^	Bostman score
1	72	M	DM^c^	Fall on the ground	R	Comminuted	66	31	8	0	0–0‐120	25
2	45	M	None	Fall on the ground	R	Comminuted	58	26	8	0	0–0‐130	30
3	45	M	None	Fall on the ground	R	Comminuted	55	26	8	0	0–0‐135	30
4	55	M	Gouts	Fall on the ground	L	Comminuted	45	24	12	0	0–0‐130	29
5	41	F	None	Fall on the ground	L	Comminuted	71	23	8	0	0–0‐140	30
6	57	F	None	Fall on the ground	R	Comminuted	77	23	12	0	0–0‐140	30
7	67	M	Hypertension	Fall on the ground	L	Comminuted	52	22	8	0	0–0‐130	29
8	66	F	Bilateral knee OA^d^	Fall on the ground	L	Comminuted	54	20	12	1	−10‐0‐125	25
9	41	M	None	Fall on the ground	L	Comminuted	59	19	12	0	0–0‐130	30
10	45	F	DM^c^	Fall on the ground	R	Comminuted	55	19	12	0	0–0‐140	30
11	42	M	None	Fall on the ground	L	Comminuted	61	18	12	0	0–0‐140	30
12	51	F	None	Fall on the ground	L	Comminuted	66	17	12	0	0–0‐135	29
13*	73	F	DM^c^; Bilateral Knee OA^d^	Fall on the ground	R	Comminuted (Gustillo I)	94	15	12	2	−20‐0‐110	20
14	64	M	Hypertension	Fall on the ground	R	Comminuted	44	14	12	0	0–0‐140	26
15**	73	F	CAD^e^	Fall on the ground	R	Comminuted	57	13	8	1	0–0‐110	24
16	53	F	None	Fall on the ground	R	Comminuted	81	12	16	0	0–0‐125	27

^a^ PI‐NRS = Pain Intensity‐Numerical Rating Scale (PI‐NRS), 0 = no pain to 10 = worst pain imaginable.

^b^ ROM = Range of motion; it is recorded as the format of maximum extension‐0‐ maximum flexion.

^c^ DM = Diabetes Mellitus.

^d^ OA = Osteoarthritis.

^e^ CAD = Coronary Artery Disease.

* It was an emergent surgery. The glucose level was not stable after the operation, and this patient suffered from acute cellulitis 3 days after the operation. Debridement and insulin pump were arranged on day 4.

** The patient underwent coronary artery stenting 5 years before the injury, and her condition remained stable after the procedure.

This study was approved by the medical ethics committee of Beijing Jishuitan Hospital (Institutional Review Board ID: 202110‐01). All the patients gave informed consent for their follow‐up results to be published for academic purposes.

### Operative Technique

#### Step 1

All the procedures were performed under spinal anesthesia with patients in the supine position. A sheet‐folded ramp was placed under the ipsilateral buttock to direct the affected patella upwards. A radiolucent bump was sterilized and put under the popliteal fossa with the knee joint flexed 15° to 30°. A padded tourniquet was routinely placed on the thigh.

#### Step 2

A midline longitudinal incision was used from the proximal margin of the patella to 2 cm proximal to the patellar tendon insertion. There was no need to expose the insertion of the patellar tendon.

#### Step 3

**FIGURE 1:**
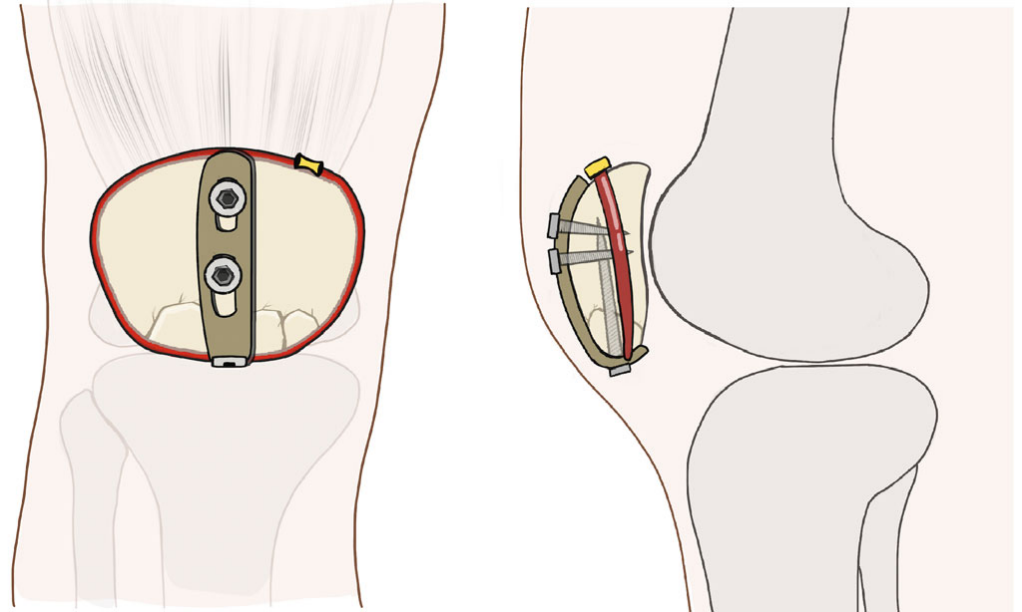
Schematic illustrations of the surgical technique

The fracture was identified and cleansed of clot and avulsed retinaculae. The radiolucent bump was moved under the ipsilateral heel to let the knee fully extend, which allowed for relaxation of the extensor mechanism and facilitated reduction of the fracture. A non‐absorbable suture (#5 Ethibond®) cerclage was prepositioned without knotting. Manipulating the suture arms helped the reduction of the fracture and prevented the inferior patella pole from fragmenting even more, which was likely to happen if done with forceps. After the continuity of the extensor mechanism was restored, the fine‐tuned reduction was performed with dental picks. Then, provisional fixation was achieved by tightening the prepositioned suture cerclage instead of a pointed reduction forceps, as the latter might not be able to effectively grasp the comminuted fragments (Figure 1).

#### Step 4

**FIGURE 2:**
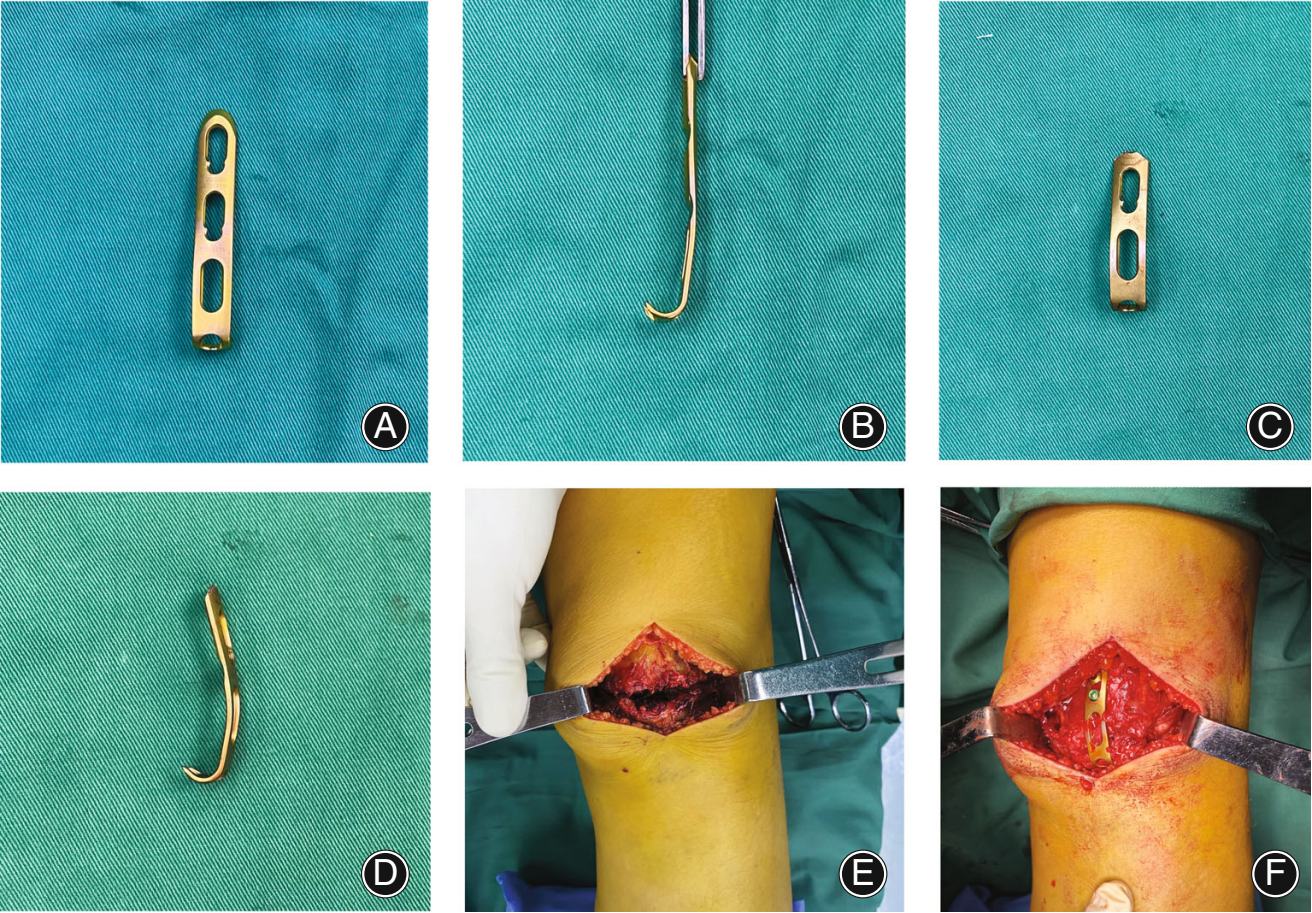
The shortest subtype of 3.5‐mm hook plates (Synthes GmbH, Switzerland) was trimmed and pre‐contoured to accommodate the length and shape of the patella (A–F)

**FIGURE 3:**
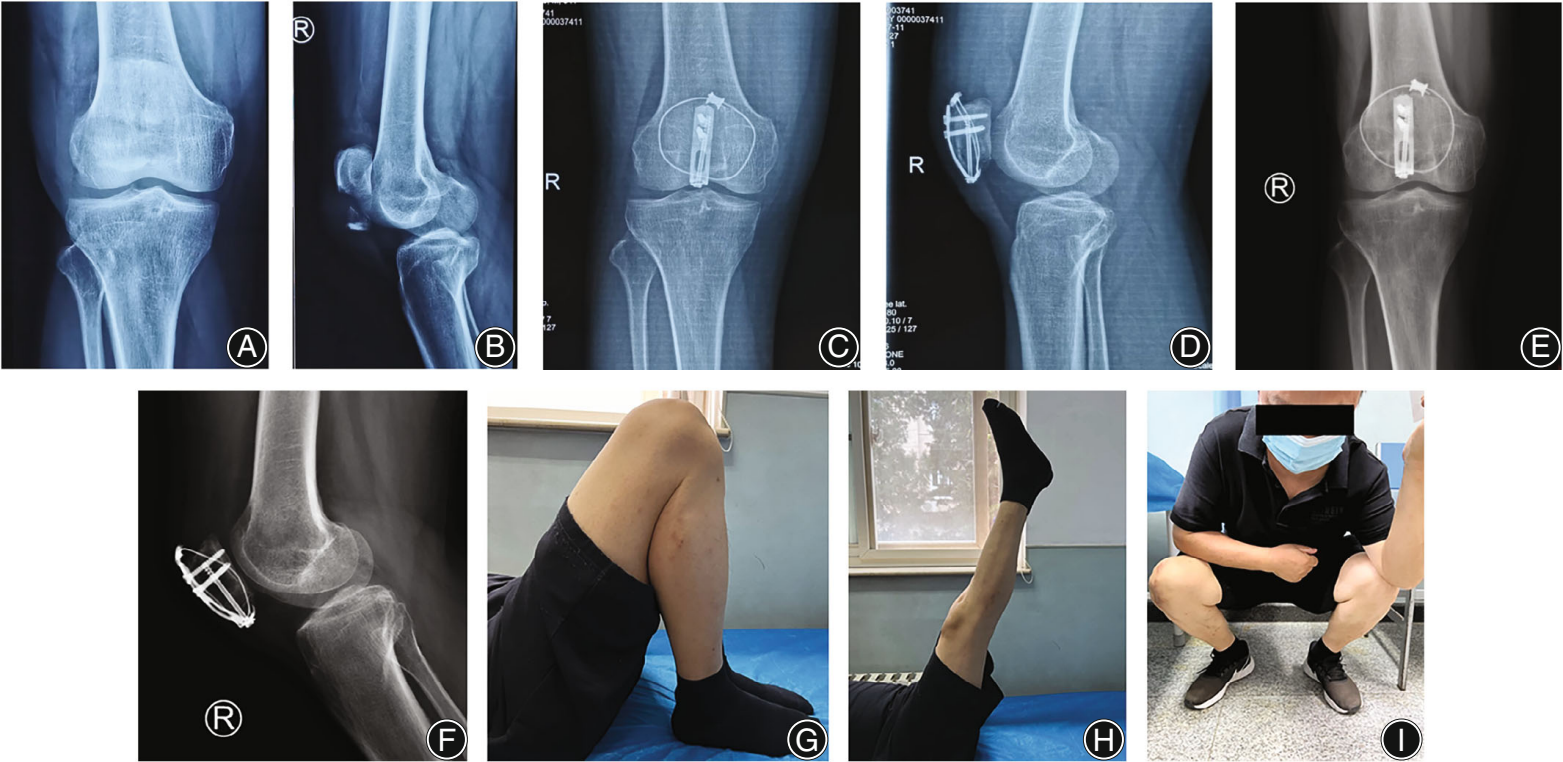
Case example (case 4, 55 years, male, right side). Preoperative anteroposterior (A) and lateral (B) radiographs showed a displaced inferior patellar pole fracture. Postoperative anteroposterior (C) and lateral (D) radiographs. Radiographs (E‐F) and function (G–I) at 2 years follow‐up

**FIGURE 4:**
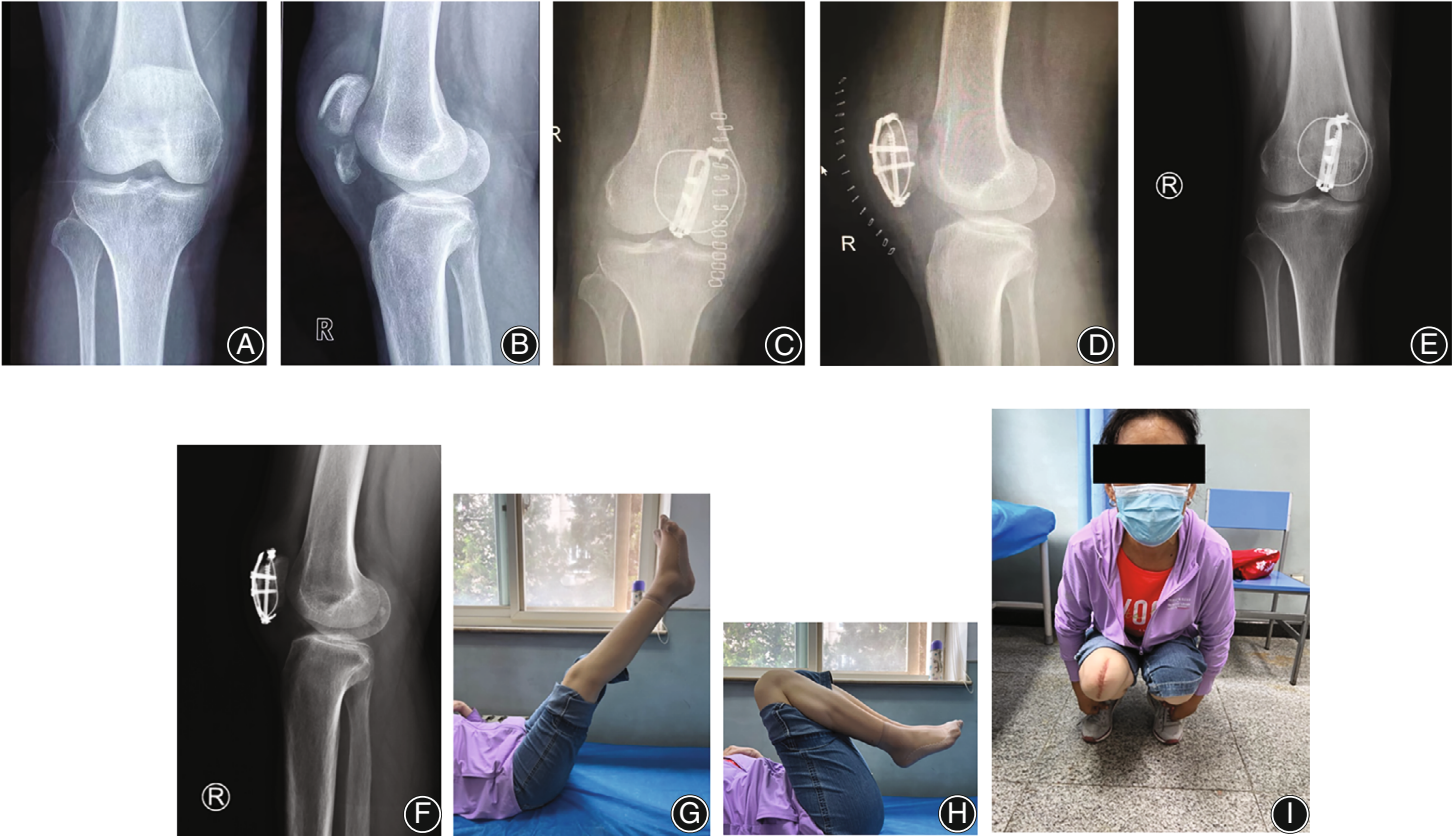
Case example (case 10, 45 years, female, right side). Preoperative anteroposterior (A) and lateral (B) radiographs showed a displaced inferior patellar pole fracture. Postoperative anteroposterior (C) and lateral (D) radiographs. Radiographs (E, F) and function (G–I) at 19 months follow‐up

After anatomical reduction confirmed by the cortical interdigitation and the intraoperative fluoroscopy, a 1.0‐mm titanium cable (Synthes GmbH, Switzerland) cerclage was guided around the whole patella by the epidural cannula. A 3.5‐mm hook plate (Synthes GmbH, Switzerland) was trimmed and pre‐contoured to accommodate the length and contour of the patella before being applied (Figure 2). The tips of hooks were then used to penetrate the patellar tendon just distal to the cable cerclage and beneath it, which prevented the laceration of the patellar tendon fibers. Next, a 3.5‐mm cortical screw was eccentrically applied to the most proximal hole of the plate using the sliding compression technique. If necessary, a 4.0‐mm cannulated half‐threaded screw was inserted from distal to proximal direction combined with a washer through the certain distal hole just between the two hooks of the plate. This was because the diameter of the distal hole of the hook plate was near the outer diameter of the cannulated screw inserted from the distal to proximal direction. Therefore, the screw would pass through the distal hole without a washer. In addition, a washer could prevent the screw head from becoming a stress riser from penetrating the comminuted inferior patellar pole and let the compression force transfer through the washer and the two hooks of the plate. It is important to note that this step was guided by fluoroscopy, which is necessary to confirm the available trajectory. The retinacular tears were repaired. Passive motion in full range was visually and fluoroscopically checked. The wound was closed in layers with absorbable sutures (#2 Vicryl®). Standard anteroposterior and lateral views of the affected knee were routinely obtained before the patient left the operation room. (Figures 3 and 4).

Prophylactic antibiotics and non‐steroid anti‐inflammation drugs were intravenously administered within 24 hours postoperatively.

### Postoperative Rehabilitation

No limitation or immobilization was necessary after the operation. Straight‐leg elevation and ankle joint contraction was initiated 1 day after surgery. Active and Passive Range of Motion (ROM) exercises (range 0°–30°) and full weight bearing were encouraged as tolerated under the help of a physiotherapist during the first postoperative week. By the end of the first month, the ROM was expected to improve to at least 0°–90° gradually.

### Follow‐Up

According to the routine schedule in our hospital, the time points for follow‐up were set as 2, 4, 8, 12, 26, and 52 weeks after the operation. Anteroposterior and lateral radiographs were obtained on day 1 and 4, 8, 12, 26, and 52 weeks after surgery.

### Outcome Measures

The time of surgery was recorded intraoperatively. Bony healing status was recorded at every follow‐up. Range of motion (ROM), PI‐NRS, and Bostman score were recorded 4, 8, 12, 26, and 52 weeks after the surgery and at the final follow‐up. All assessments were obtained by the same assistant who was blinded to the surgical procedure and clinical outcomes.

### Fracture Healing

Fracture healing was defined as a radiologically translucent line disappearance between fracture sites within 6 months.

### Pain Intensity‐Numerical Rating Scale (PI‐NRS)

PI‐NRS is a visible analog scale ranging from 0 (no pain) to 10 (most severe pain ever imagined). It is categorized as no pain (0), mild pain (1–3), moderate pain (4–6), and severe pain (7–10).

### Bostman Scores and Range of Motion (ROM)

Bostman scores were used to evaluate functional knee outcomes,^20^ and ROM in degrees was assessed by goniometry. The overall 30‐score Bostman score scheme consists of ROM (6 points), pain (6 points), work (4 points), atrophy (4 points), ambulation assistance (4 points), effusion (2 points), giving away (2 points), and stair climbing (2 points). A total score of 30–28 is considered as an excellent result, 27–20 is considered a good result, and <20 is an unsatisfactory result.

## Results

### General Results

All the injuries were caused by falling on the ground with flexed knee, and all the fractures had comminuted patterns in CT scans. The mean follow‐up period was 20.1 ± 5.3 months (range 12–31), and the cases in this cohort were followed up once during July to September 2021. The average time of surgery was 62.2 ± 13.3 minutes (range 44–94).

### Fracture Healing

All the fractures healed well, within an average of 10.8 ± 2.4 weeks (range, 8–16 weeks). Also, no hardware irritation or failures have been reported.

### Pain Intensity‐Numerical Rating Scale (PI‐NRS)

At the last follow‐up, 12 patients (75%) reported no pain (PI‐NRS score of 0), and the remaining four patients (25%) reported mild pain (three with a PI‐NRS score of 1 and one with a score of 2).However, all the patients who reported pain had osteoarthritis of bilateral knee joints and did not report exacerbation (which was present before the operation).

### Bostman Scores and Range of Motion (ROM)

The average Bostman score was 27.8 ± 3.0 (range, 20–30), and all the patients showed excellent or good results. The average ROM was 127.5 ± 13.9° (range, 90°–140°).

### Removal of Implants

Eleven patients (68.8%) requested the removal of implants for individual reasons, such as the convenience of passing the security scans, undergoing MRI examination, or some religious reasons. No patient reported hardware induced irritation.

### Complications

At 16 weeks post‐surgery, patient 16 consulted the hospital in order to resolve weakness and stiffness of the affected knee. Radiographs showed bone healing; the patient was prescribed a personalized rehabilitation program. Good results were seen at week 26.

Flexion contracture was found in case 8 and case 13, both of which had OA and no exacerbation compared with the outpatient medical documents before the fractures.

Acute cellulitis was found in a 73‐year‐old female patient with Gustillo I open fracture caused by falling onto the knee. Debridement and definite internal fixation were performed in the emergency operation theater on the day when the accident happened. The glucose level was not stable after the operation, and acute cellulitis appeared 3 days later. Debridement with copious 0.9% saline irrigation was performed on day 4, without removing the implants. During the following inpatient period, the inflammation subsided, and the glucose level was normalized under the control of an insulin pump. Also, the patient suffered from osteoarthritis of bilateral knee joints for nearly 20 years, which was also why she complained about pain around the knee joints. Because the pain was symmetrical and was not exacerbated postoperatively, it was not considered as secondary to the operation.

## Discussion

Partial patellectomy was once considered as a surgical treatment approach for the comminuted inferior patellar pole fracture. Yet, recent data suggested that partial patellectomy provides poor results.^5^, ^6^, ^7^

Bone‐preserving procedures are commonly used in current clinical work. Yet, those procedures may be challenging and complex. First of all, the quadriceps is one of the most powerful muscles in the human body. Zernicke*et al*.^21^ reported that the quadriceps could support a contractile force of more than 17.5 times body weight. Secondly, the inferior patellar pole fragment is always of small size with comminution. Moreover, there is no universal standard that could be used for the guidance and surgical decision‐making in this kind of case. Thus, surgeons have to rely on their clinical judgment to guide the treatment for inferior patellar pole fractures. The chief goal of our method is to restore the continuity of the extensor mechanism with enough stability to resist the contractile force of quadriceps in early rehabilitation. Biomechanical testing with transverse patellar fracture models showed that plates have an even better treatment effect compared to tension band techniques.^14^, ^15^, ^16^ The plate construct demonstrated the lowest amount of fracture gapping under cyclic load compared with either Kirschner wire or cannulated screw tension band constructs.^22^ Thus, plating could be considered as an ideal option to resist the strong tension of the extensor mechanism. On the other hand, the inferior patellar poles are often small and comminuted, so it would be better to prevent too much insertion of implants from this small fragment. Adding a cable cerclage to enhance the stability seems to be an effective method.^8^

### Technical Features and Strengths

Theoretically, the stability is provided by a cable cerclage in the coronal plane and the hook plate in the sagittal plane. Based on our findings and previously published study,^3^ a substantial rate of inferior patellar pole fractures has a comminuted pattern with a small size, which might be problematic for some of the current techniques. For example, the comminuted inferior patellar pole might be lacerated using several separate vertical wirings.^23^ As a cable was used to hold the fragments of the inferior patellar pole together as a whole, it could be easily compressed by the hook plate. Besides, the hooks of the plate were inserted beneath the cable, which could prevent the hooks from cutting through the comminuted inferior patellar pole. Therefore, the first feature of our technique is the effective fixating of the comminuted fragments. The second feature of our procedure is less damage to the extensor mechanism and the around soft tissue. Compared with tension band technique, this procedure does not require the quadriceps tendon to be split. The sharp edge or tip of implant would not be exposed to the extensor mechanism during motion, so our technique avoids the problems that are common in tension band technique, such as irritation induced by ends of Kirschner wires and implant migration. Also, only two penetration holes were made in the patellar tendon for the insertion of the plate hooks. Zhu *et al*. introduced a method based on a combination of a miniplate with a tension band, whose possible drawback was a dissection of the patellar tendon for the plate placement.^9^ Considering the small size of inferior patellar pole fragment, it is necessary to maintain the bony volume as much as possible. However, for the fixation with miniplate augment,^9^, ^10^ basket plates,^13^ or cannulated screws,^24^ bone loss is inevitable in the case of multiple screws placement through the small inferior patellar pole fragment. In addition, the specific basket plates are not available in all the institutions, while a hook plate can even be made at home by cutting a 1/3 tubular plate^19^ or a reconstruction plate.^18^ Thus, the third feature of this method is reproducibility and ease of propagation, as well as maximal maintenance of the bone volume.

### Effectiveness and Safety

Early rehabilitation was immediately initiated on day 1 postoperatively in regular patients, and full weight bearing was not limited in patients managed by this procedure. All the patients achieved the bony healing without secondary displacement of implant failure, as well as excellent or good results confirmed by Botsman score at final follow‐up. Although complications occurred in two cases, they were not due to fixation procedures or implants. Patient 13 (Gustillo I open fracture) accepted the debridement procedure on day 4 postoperatively due to the ongoing acute cellulitis, which was controlled by the empirical intravenous antibiotics. The inflammation subsided after surgical management. Patient 16 was non‐local resident, so she did not receive regular rehabilitation, and she missed the first three postoperative months of follow‐up. She was re‐admitted as an outpatient at 4 months postoperatively complaining of weakness during full weight bearing walking and stiffness when flexing over 90°. As the bony healing was confirmed, individual aggressive rehabilitation was urged under the supervision of the physiotherapist. After 3 months, the patient's condition improved, and good results were found at the time point of 26 weeks follow‐up. Excluding the above‐mentioned adverse events, no other complication was found, such as implant migration, breakage, or irritation. In fact, implant associated complication has been a problem for orthopaedic surgeons. For example, Lazaro *et al*. reported a rate of 37% hardware removal due to prominent and symptomatic implants as a result of breakage or continuous soft tissue irritation.^25^ Greenberg *et al*. reviewed 44 patients who underwent implant removal following tension band fixation and found 33 (75%) of 44 patients had less pain after removal of implants. The explanation of no implant‐induced pain in our cohort might be no metallic sharp end exposed, such as tips of Kirschner wires or end of stainless wire. Therefore, it is a method with satisfactory effectiveness and reliable safety.

### Limitations

Following are some limitations in the present study: (i) a retrospective design and small case numbers; (ii) no comparison to other treatment options; (iii) absence of long‐time follow‐up. In addition, the 3.5‐mm hook plate (Synthes GmbH, Switzerland) is not primarily intended for patellar fractures. Even the shortest sub‐type was too long, so it had to be trimmed, and pre‐contoured to match the length and shape of the patella.

### Conclusion

A combination of cable cerclage and hook plate is a reliable and stable fixation construct for managing inferior patellar pole fractures. With easily obtained devices and minimal injury to the tendon, rigid stability can be achieved to promote early rehabilitation. A prospective cohort study with longer follow‐up should be conducted to demonstrate the pros and cons of this technique.

## Conflict of Interest Statement

There is no conflict of interest related to this work.

## Author Contributions

Hangyu Gu: First author. Designed the study and the surgical technique. Operator of the surgeries. Collected and analyzed the data. Wrote the manuscript.

Xinbao Wu: Corresponding author. Designed the study. Analyzed the radiographs and follow‐up data. Wrote the manuscript. Gave the final approval of submission.

Ting Li: Collect the data. Wrote the manuscript.

Shiwen Zhu: Collect the data. Wrote the manuscript.

## Declaration of Interest

There is no conflict of interest related to this work.

## Funding Statement

No benefits in any form have been received or will be received from a commercial party related directly or indirectly to the subject of this article.
